# Empowering tomorrow’s public health researchers and clinicians to develop digital health interventions using chatbots, virtual reality, and other AI technologies

**DOI:** 10.3389/fpubh.2025.1577076

**Published:** 2025-07-08

**Authors:** W. Scott Comulada, Catherine McQueen, Cathy M. Lang

**Affiliations:** ^1^Department of Psychiatry and Biobehavioral Sciences, Semel Institute Center for Community Health, University of California, Los Angeles, Los Angeles, CA, United States; ^2^Department of Health Policy and Management, University of California, Los Angeles, Los Angeles, CA, United States; ^3^Department of Emergency Medicine and Informatics, Institute for Prediction Technology, University of California, Berkeley, Berkeley, CA, United States; ^4^Department of Community Health Sciences, University of California, Los Angeles, Los Angeles, CA, United States

**Keywords:** digital health intervention, health communication, chatbot, virtual reality, artificial intelligence

## Abstract

**Background:**

Artificial Intelligence (AI)-based digital health interventions incorporating technologies like chatbots and augmented/virtual reality are reshaping the healthcare delivery landscape. The rollout of these technologies warrants updated graduate curricula to train future healthcare professionals. In response, the authors incorporated additional topics relevant to digital health intervention development into a graduate-level digital health communication course and evaluated student feedback.

**Methods:**

The authors developed four lectures on two−/one-way digital health messaging strategies, AI/large language models, chatbots, and augmented/virtual reality, and a chatbot development tutorial as a lab. They evaluated students’ perceptions of the course and the benefits of the new content after course completion through standard and supplemental course evaluations.

**Results:**

Eleven of 16 enrolled students completed the course evaluation, and 8 completed the supplemental survey. Most students were from the school of public health and reported female gender. One of 8 students completing the survey reported prior experience creating chatbot and AR/VR content. The overall average course rating was high (7.45 out of 9). Open-ended survey responses about the new content were mixed with enthusiasm and questions about its relevance over content on traditional communication modalities in preparation for public health work.

**Conclusion:**

Student feedback underscored course content value, along with guidance to better emphasize how chatbots and augmented/virtual reality are relevant to clinical and public health practices. More applications relevant for diverse populations could elucidate the value of new technologies for students who will develop digital-based interventions. Applications focusing on commonalities could also solidify students’ understanding of intervention development principles that will remain, as technologies evolve.

## 1 Introduction

Within the past 5 years, two events have profoundly altered healthcare delivery and, in turn, the need for educational institutions to update curricula for future healthcare researchers and providers: the emergence of COVID-19 and the generative pre-trained transformer (GPT) (1), a form of generative artificial intelligence (AI). The COVID-19 pandemic accelerated the adoption of telehealth services that use digital communication technologies to overcome barriers to care imposed by social distancing, overburdened healthcare systems, and healthcare worker shortages (2, 3). Telehealth services incorporate GPTs and other AI technologies to better support healthcare delivery, e.g., to automate patient monitoring and communication (4).

Interventions designed to address health conditions through digital technology, hereafter referred to as digital health interventions, are also embedded in telehealth services and have benefited from AI advances (5–7). For example, text messages (SMS) serve as a medium for health promotion content, surveys, automated medical appointments, and medication reminders (8–10). Through AI, just-in-time adaptive interventions (JITAI) use mobile phone sensors and ecological momentary assessment to tailor SMS content and intervene in the moment when needed (11). Conversational agents, or chatbots, especially those using ChatGPT and other large language models (LLMs), enhance SMS communication by allowing patients to converse with healthcare computer systems to better meet their needs (12–14). Clinicians and researchers are also incorporating ChatGPT and LLM into augmented and virtual reality (AR/VR) scenarios to create embodied conversational agents, such as avatars that converse with patients and exhibit human expressions on their virtual faces (15). Xaia is a robot avatar and virtual health coach that patients can view and converse with through the Apple Vision Pro headset to receive mental health support between visits with a healthcare provider (16). In addition to patient-facing interventions, clinicians are developing AI-driven simulations for clinical students to bolster communication skills training and ultimately improve patient care (17, 18).

Given the rapid advances in AI-related software and AR/VR hardware, there is a growing gap between interventions that clinicians and public health researchers are developing in the field and learning how to develop and evaluate in school (19). An increasing body of literature highlights the use of AI and chatbots to improve classroom instruction (20) and a growing number of undergraduate and graduate degree programs are using AI and AR/VR tools to help students visualize anatomy and medical procedures, such as the University of California, Los Angeles (UCLA) Simulation Center and New York University’s Education Digital Experience (EDX) program. There are also programs, such as UCLA’s Master of Data Science in Health (MDSH) degree, that provide instruction on health informatics and data science and teach students how to harness AI tools and analyze data from computational systems like electronic health records. What is missing is training for future clinicians and researchers to learn about AR/VR hardware and chatbot applications and how to use them to develop and deliver AI-based digital health interventions for health education and promotion. Given the dangers and limitations of AI, e.g., those that can exacerbate biases and discriminatory practices toward under-served populations (21, 22), there is also a need for instruction on how to evaluate the appropriateness of AI technologies for healthcare and public health settings.

To date, there is a proliferation of LinkedIn and other online course content that covers chatbot and AR/VR software development. Fewer undergraduate and graduate degrees offer similar course content, especially in the health sciences, with the Cedars-Sinai Master’s Degree in Health Delivery Science (MHDS) program as an exception. As faculty in schools of medicine and public health, we have observed that our students mostly learn about intervention development through standard public health degrees, often an MPH or PhD. It’s important for school programs to provide instruction on intervention development in the context of emerging AI technologies that are becoming commonplace in clinical and public health settings. For example, healthcare organizations are using chatbots to provide remote health services to patients that would otherwise be impractical due to healthcare worker shortages (23). Research has demonstrated the benefits of VR therapy to treat mental health disorders (24) and help patients manage pain (25). Therefore, we advocate that public health curricula need health communication courses that introduce students to chatbots, AR/VR, and other AI digital health intervention tools.

To address this need, we developed new course material focused on chatbots and AR/VR as emerging digital health intervention tools within an existing health communication course that also provides foundational theories and techniques to create effective health content. The course was offered through a community health sciences department within a school of public health. We chose this course because it covered digital formats for health education and promotion. The university provides an anonymous course evaluation for students to fill out. Since the content was new, we administered an additional evaluation to gage the fit of the new content for this course, its perceived benefits to help students prepare for public health careers, and suggestions for future course modifications. We hypothesized that students would perceive the benefit of the new course content because of the growing use of chatbots and AR/VR in health interventions.

## 2 Materials and methods

### 2.1 Course overview

This study evaluated an elective four-unit graduate health communication course offered at the UCLA. This course emphasizes student mastery in understanding how the public uses news media and digital information technology to obtain health information and how public health educational materials can incorporate these delivery platforms. Students must take an introductory community health sciences course or an equivalent social sciences course as a prerequisite. No prior experience using digital information tools is necessary to enroll.

### 2.2 Course content and format

There are five primary objectives for students in the course: (1) design messages for health communication for different target populations, (2) learn to use new digital technologies for health communication production and distribution, (3) obtain an overview of basic principles of communication with different populations, (4) integrate theoretical perspectives into communication strategies and materials, and (5) understand access to and utilization of informatics and communication technologies among professional and general public populations. The course meets in-person weekly for 10 weeks. Instructors deliver course content through lectures over the first 2 h of each weekly session and conduct computer labs for the last hour for approximately half of the weekly sessions that introduce students to freely available software programs they can use to develop health promotion material, including GIMP, Scribus, Audacity, Shotcut, and WordPress. Table 1 summarizes lecture topics and lists software packages covered in computer labs. The course introduces students to health promotion theories and practices for developing equitable health promotion content and interventions, e.g., codesign principles for developing and pilot testing interventions with intended populations to determine feasibility before full deployment. The course also provides tutorials on the software described above, fosters class discussions about health promotion strategies and technologies, and includes assignments that guide students through the development of health education/promotion materials. Materials range from health promotion calendars/booklets to podcasts, videos, and social media campaigns. Students showcase their materials through a class presentation in the last (10th) week.

**Table 1:** Fifteen lecture topics and six software packages covered during lab sessions over 10-week graduate health communication course.

Lecture topics by week	Lab sessions
1a: Introduction to health communication, information technology for health promotion and communication, and digital literacy 1b: How people learn, message design theories	
2: Formative research methods to design health messages (e.g., focus groups, in-depth interviews)	GIMP, image editing program https://www.gimp.org/
3a: Tools (e.g., message maps) and considerations for developing health promotion messages (e.g., readability and numeracy) 3b: Video, podcasts, and website formats for health communication	Audacity, audio editing and recording application https://www.audacityteam.org Shotcut, video editing application https://shotcut.org
4a: Traditional and digital forms of health communication with a focus on traditional forms (booklets, infographics, and photonovellas) **4b: One-way and two-way forms of digital communication**	Scribus, desktop publishing software https://www.scribus.net/
5a: How to use social media platforms (e.g., Facebook, Instragram, and Twitter/X) for health communication 5b: Video, podcasts, and website formats for health communication	WordPress, website creation and web content management system https://wordpress.com/
**6: Chatbots and other AI-driven digital communication tools**	**Botpress, chatbot (AI agent) creation platform** https://botpress.com/
7a: How people seek health information on the Internet and their characteristics **7b: How digital tools are changing clinical education (e.g., virtually simulating clinician-patient encounters)**	
8a: From toolkits to texting to apps and wearables for public health **8b: Virtual reality, avatars, games and health**	
9a: Popular media and transmedia 9b: Long and short documentaries	
10: No lectures (Class presentations)	

New lecture topics and the new lab session are bolded.

### 2.3 New course content

**Figure 1:**
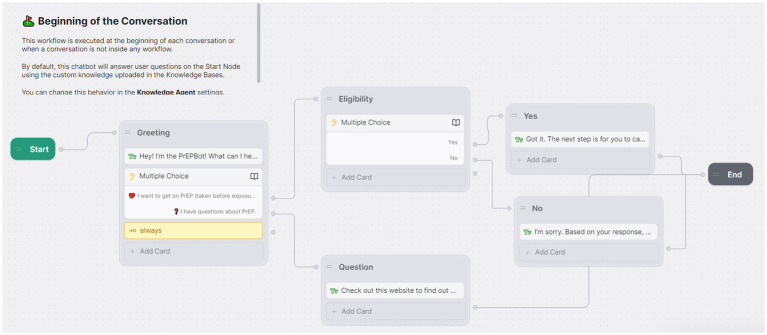
Screenshot of Botpress graphical user interface showing conversation flow that students constructed during their chatbot lab.

As illustrated in Table 2, we used a logic model to guide the addition of new course material focused on chatbots and AR/VR, which is the basis for this study (26–28). We began by assessing the need for new course content by emailing seven faculties in our professional network at different institutions who develop health interventions and have school of public health appointments. In the email, we asked them if they knew of courses on digital health intervention development and saw a need for this type of course. Correspondence established a need, best summarized by one of the email recipient’s responses. “I was a learn this on-the-fly person, but developing courses in this area sounds amazing.” Supplementary Table 1 lists email responses from all seven faculties. We delivered new content through lectures across four topics: (1) one-way versus two-way health communication in order to distinguish static interventions from those which encourages interactivity; (2) AI and LLM concepts suitable for students with limited statistical backgrounds; (3) chatbots; and (4) AR/VR. Additionally, we developed a new lab where students programmed a chatbot using Botpress, an AI chatbot development platform that lets users access basic development tools for free. The chatbot lab was based on a real-world scenario relevant to health department HIV prevention efforts. We tasked students with developing a simple version of a chatbot that would allow clients to text message the chatbot to inquire about pre-exposure prophylaxis (PrEP; Figure 1).

**Table 2:** Logic model used to plan and evaluate additional content added to existing health communication course on emerging digital tools (i.e., chatbots and AR/VR) that are being used to develop interventions.

Inputs	Activities	Outputs	Short-term outcomes	Long-term Outcomes
Intervention developers Faculty Schools of public health Instructor existing health communication course	Curriculum development and delivery Describe how digital tools enhance existing interventions Examples of digital tools Illustrate how to use digital tools Generate urgency to learn about digital tools to enhance students’ future work in public health fields	Lectures on four topic areas Two- vs. one-way communication Primer on AI Chatbots AR/VR	Student perceptions of added value for new course content based on standard course evaluation and supplemental evaluation administered immediately after completion of course	To be measured Actual value of new content based on feedback from graduates who are in the field and developing health interventions

### 2.4 Study design and population

We obtained qualitative and quantitative study data from two web-based optional surveys sent to all 16 enrolled students at the end of the Winter 2024 quarter: the university administers a course evaluation at the end of each quarter, and we administered a supplemental evaluation to students through an email invitation. The responses to the course evaluation were anonymous, while the supplemental evaluation responses were not anonymous to allow for participant compensation, i.e., $30 Amazon gift codes. The Institutional Review Board (IRB) at the UCLA determined the study to be exempt from IRB review #24-000551. The South General Institutional Review Board (IRB) at the UCLA waived the need of informed consent.

### 2.5 Measures

The course evaluation administered by the university contained both Likert scale questions (e.g., “To what extent do you feel that you have learned something you consider valuable?”) and a free-response question (e.g., “Please identify what you perceive to be the real strengths and weaknesses of this instructor and course.”) about the course and instructors. The course evaluation did not contain questions about sociodemographic characteristics.

The supplemental evaluation contained 25 multiple-choice questions (e.g., questions about sociodemographic characteristics and interest in the subject after taking the course) and 7 free-response questions (e.g., “What suggestions do you have for how the lectures could be improved?”) from other program evaluations we have conducted. The questions ranged from asking for feedback about the course (e.g., if there were topics students would have liked covered “in greater or less detail”) to inquiring about previous experience with various software programs, technology, and social media relevant to the field of public health.

### 2.6 Data analysis

We used descriptive statistics to summarize student demographic characteristics and quantitative survey responses. We calculated means and standard deviations for continuous measures and proportions and sample sizes for categorical measures. We used a mixed-methods approach to analyze quantitative and qualitative data to evaluate how quantitative course ratings aligned with qualitative responses to open-ended questions. Co-authors transcribed responses to open-ended questions and organized qualitative content using a grounded theory analysis framework to identify themes (29).

## 3 Results

### 3.1 Participant characteristics

Eleven of 16 enrolled students (68.75%) completed the course evaluation, and 8 students (50%) completed the supplemental evaluation. The number of enrolled students was comparable to class sizes for the other 17 graduate classes offered by the same department for the Winter 2024 quarter (median = 14 students; range = 3 to 51). Table 3 presents the demographic and background characteristics of the students completing the supplemental evaluation. Most students reported female gender (87.5%), with one student reporting non-binary gender. Students varied by ethnicity and race. Most students were within the Department of Community Health Sciences (75%), with one student from the social sciences and another from the school of nursing. Most students who completed the course evaluation were also in the department of community health sciences, except for two students already discussed above and a student from the school of medicine.

**Table 3:** Demographics and background characteristics of students who completed the supplemental evaluation (*N* = 8).

Characteristic	*n*	%
Gender		
Male	0	0
Female	7	87.5
Non-binary	1	12.5
Race		
Asian	1	12.5
Multiracial	3	37.5
White	2	25
Unknown	2	12.5
Department or School in Attendance at UCLA		
Community Health Sciences	6	75.0
Nursing	1	12.5
Social Science	1	12.5
Age		
20–25	1	12.5
25–30	6	75
30+	1	12.5

We did not have access to the demographic information for the 16 enrolled students. We only report that observed gender based on enrolled students’ preferred pronouns and appearances suggested the observed gender of all students to be female, except for one student identifying with non-binary gender, matching the self-identified gender composition for students completing the supplemental evaluation.

### 3.2 Technology experience before taking the course

Students generally had limited technical experience with digital technologies before the course, except for social media, as summarized in Table 4. Seven students (87.5%) reported no previous experience creating chatbots, while 1 (12.5%) student rated their experience level as beginner. Four students (50%) rated their experience level viewing AR/VR content through a headset as beginner, while 4 participants (50%) reported they had never viewed AR/VR through a headset before. When participants were asked about their experience level creating AR/VR content through development platforms (e.g., Unity), seven participants (87.5%) indicated they had never created virtual or augmented reality content before, and one person (12.5%) rated their experience as beginner.

**Table 4:** Students’ experience levels with software programs, technology, and social media before enrolling in course (*N* = 8).

Supplemental evaluation question	No experience		Beginner		Intermediate		Advanced	
	n	%	n	%	n	%	n	%
What was your experience level creating or manipulating audio files in software (e.g., Audacity) before the course?	1	12.5	6	75	1	12.5	0	0
What was your experience level creating chatbots in software (e.g., Botpress) before the course?	7	87.5	1	12.5	0	0	0	0
What was your experience level using a desktop publishing software program (e.g., Scribus) before the course?	1	12.5	6	75	0	0	1	12.5
What was your experience level using an image editing software program (e.g., Adobe Photoshop) before the course?	1	12.5	5	62.5	2	25	0	0
What was your experience level creating podcasts before the course?	4	50	3	37.5	1	12.5	0	0
What was your experience level using social media (e.g., Instagram) before the course?	0	0	1	12.5	2	25	5	62.5
What was your experience level creating or editing videos before the course?	1	12.5	4	50	2	25	1	12.5
What was your experience level viewing virtual reality or augmented reality content through a headset (e.g., HTC VIVE) before the course?	4	50	4	50	0	0	0	0
What was your experience level creating virtual or augmented reality content through development platforms (e.g., Unity) before the course?	7	87.5	1	12.5	0	0	0	0
What was your experience level creating websites using software programs (e.g., WordPress) before the course?	4	50	3	37.5	0	0	1	12.5

Students came into this course with a range of experience levels and backgrounds, which allowed students to build off the knowledge of others. Some students expressed that course content was approachable and beginner-friendly, even with limited digital technology experience.

### 3.3 Overall course experience

Though there were differences in experience levels and interests between students before taking the course, they entered the course interested in learning about the intersection of information technology and public health. As displayed in Table 5, the average interest in the subject before taking the course was measured at 2.36 out of 3 (SD = 0.50). After completing the course, students’ interest in information technology for health promotion and communication slightly increased; participants reported an average interest in the subject at 2.45 out of 3 (SD = 0.52). The overall course rating was similarly high, with students giving it an average score of 7.45 out of 9 (SD = 1.57). For comparison, the average score of 7.45 was similar to the average of 7.51 (SD = 1.52) across the overall course rating averages of the other 17 courses offered by the same department for the Winter 2024 quarter.

**Table 5:** Students’ course evaluation responses to Likert scale questions (*N* = 11 for all questions, except *N* = 10 for “texts, required readings” question).

Course evaluation question	*M*	*SD*
What was your interest in the subject before taking CHS 292? (1 = Low, 3 = High)	2.36	0.50
What was your interest in the subject after taking CHS 292? (1 = Low, 3 = High)	2.45	0.52
To what extent do you feel that you have learned something you consider valuable in CHS 292 (1 = Very low, 9 = Very high)	7.55	1.29
What is your overall course rating? (1 = Very low, 9 = Very high)	7.45	1.57
Mastery of course material (1 = Low, 3 = High)	2.36	0.50
Difficulty relative to other courses (1 = Low, 3 = High)	1.91	0.54
Workload/pace was (1 = Too slow, 3 = Too much)	2.18	0.40
Texts, required readings (1 = Low, 3 = High)	2.30	0.48
Homework assignments (1 = Low, 3 = High)	2.55	0.52
Lecture presentations (1 = Poor, 3 = Excellent)	2.45	0.69
Class discussions (1 = Poor, 3 = Excellent)	2.27	0.65

### 3.4 Course content feedback

Students reported feeling confident in their mastery of the course content, with their self-reported mastery of the course material averaging 2.36 out of 3 (SD = 0.50). As the course content covers different topics at the intersection of information technology and public health (e.g., health promotion social media posts and podcasts), students appreciated the breadth of topics and software programs covered in the course. This sentiment was best expressed by one student’s comment: “There was a lot of topics covered and great resources included in the lectures that I found very useful referencing later in the class as I was working on the projects.” Supplementary Tables 2–5 present all the students’ comments from the supplemental evaluation. Interest in the content extended to discussions about chatbots and AR/VR as emerging technologies. One student expanded on this, explaining they would’ve loved to learn “more about chatbots – this is an up an[d] coming area.” Students also noted these more novel technologies as interesting and exciting to learn about, particularly after seeing how they can be applied in a public health setting. Appreciation for course content aligned with course evaluation results. Students reported a high perceived value in the course’s learning outcomes, with an average rating of 7.55 out of 9 (SD = 1.29). Students’ ratings for required readings were high, with an average score of 2.55 out of 3 (SD = 0.52). Lecture presentations were rated with an average of 2.45 out of 3 (SD = 0.69).

Amidst interest in chatbots and AR/VR, some students felt that learning about these newer forms of digital technology may have taken time away from learning about other course content or strayed from the core of the class. To show, one student expressed an interest in VR but showed hesitation about its appropriateness for the course, writing, “It was exciting to see how these new tools like VR are being translated into the healthcare space. Would have really enjoyed exploring some of those tools more, though it might be out of scope for the class.” Along this same line, when providing feedback on what they would change about the content, another student stated, “Chatbot development was interesting, but maybe not the most valuable. Since there is such limited class time I would maybe offer that as a supplemental resource and not have it take up class time.” Some students wanted more class time devoted to providing more information on best practices for communicating with diverse populations, though the course touches on this topic. For example, one student noted that they were left wishing they had learned more about “forms of media for populations [that] have accessibility issues (deaf, blind, etc.) and how we can reach these populations through health communication.”

### 3.5 Career preparation

Students also provided insights on the applicability of aspects of the course content to their future careers. Students noted that the resources presented and exposure to digital technologies helped them develop practical skills that they can apply in the future. One student touched on this, writing, “I liked that we learned real skills, and the printed handouts and additional resources were really great to refer to, and are something I will hold onto for future use.” Additionally, some students felt that the selection of digital technologies covered in the course could be changed to prepare them for their careers better. One student touched on this, stating that they wish the course taught, “more updated and user friendly platforms. ShotCut, [A]udacity, Scribus, WordPress all felt outdated and not very easy to navigate. I think learning Adobe Photoshop, Canva, and other website platforms would have been more helpful and applicable (because i have heard of PH orgs using these platforms in the real world).” Students also expressed that they wished the course spent more time on basic design skills with conventional media to prepare them for their future roles. One student commented on this, writing, “Learning skills and principles with more conventional media make more sense for a foundational class like this, and are more useful for people looking to work on health communication materials for work. I understand that it is interesting to talk about new technologies, but learning how to make a quality brochure or website is a more marketable and applicable skill than learning about VR.”

## 4 Discussion

This study evaluated students perceived benefits about new course content on AI-driven health intervention technologies, including chatbots and AR/VR, that the authors added to a graduate-level health communication course in a school of public health. Mixed findings highlighted a spectrum of perceived benefits from students who were enthusiastic about the new course content to those who were not interested in it; there were students in between who wanted clarification as to its relevance for public health students. Variation in perceived benefits for learning about AI-driven health intervention technologies reflect variations in other student populations, e.g., medical students (30), and the public regarding technology adoption from “innovators” and “early adopters” to “laggards” (31).

It is important for public health educators to cultivate the enthusiasm toward technology that students who are the “innovators” and “early adopters” bring to the classroom. These students can help engage other students in new technology-focused course content and be inspired to lead technological development as future health professionals, a role that will be needed amidst a digital healthcare transformation that is underway (32, 33). Specialized courses on emerging public health innovations will be one way to accommodate “innovators” and “early adopters.” For example, the University of Michigan School of Public Health offers a “ChatGPT and Public Health” course (34).

In addition to new courses, student feedback indicated a pathway forward to incorporate new content into existing public health courses that could appeal to “innovators” and “early adopters.” Public health educators can offer supplementary course content for students who want more comprehensive coverage of certain topics. For example, we offered a single lab on chatbot development. One student suggested that the class offer an additional lab on chatbot development illustrating different software. This could have been accomplished through supplementary lab material. Between chatbots and AR/VR as the two emerging technologies we focused on, student feedback indicated that they saw greater relevance for chatbots in public health. This may be due in part to hands-on lab experience developing a chatbot that solidified public health applications we covered during lectures. We did not have a lab on AR/VR development due to the added complexities of the development process that involves visualizations, relative to the simple graphical user interface to develop text-based chatbot dialog, as depicted in Figure 1. In retrospect, we could have allocated more time to develop an introductory AR/VR lab. Some software packages have free versions suitable for introductory labs that would allow students to prototype [e.g., Figma (35)] and develop AR/VR experiences without writing code [e.g., HyperSkill (36)]. HyperSkill has a healthcare focus with pre-built avatars and scenes, including clinician and patient avatars and medical facilities that support healthcare AR/VR experiences.

A challenge in teaching an introductory course like this one is that it is intended to expose students to different digital technologies useful for health campaigns and interventions. As a result, some students received the course material better than others depending on experience levels with the digital technologies that students came into the course with, as well as the range of desired career trajectories. Students had varying opinions on which digital technologies should be prioritized and added insight into a possible solution to cover more technologies and software options by offering supplemental lab material to cover outside of the classroom. Minimizing course expenses while introducing students to industry-standard software they may use after graduating is also challenging. We utilized free programs for continual usage after course completion so that we could introduce multiple software packages to students. However, some students expressed an interest in learning to use commercial software like Adobe photograph editing and desktop publishing software versus GIMP and Scribus, respectively. Another consideration is that free versions of Botpress and other software to develop chatbots and AR/VR applications allow for exploration suitable for an introductory lab but require payment beyond that, which may be cost-prohibitive for more extensive use. Additional emphasis should be placed on understanding what these software programs can do and how learning the skills from these free programs is translatable to commercial versions. Moreover, this course highlights effective health messaging techniques, which are a cornerstone of intervention design and a skill that can be translated across all digital and non-digital media formats.

Students also provided helpful feedback for engaging students who may be later adopters of new technologies. Amidst our presentations on emerging digital technologies and software packages, we emphasized long-standing health promotion and intervention development principles. Class assignments required students to specify conceptual frameworks for health messaging, intended populations, the health needs of the population, and how chosen technologies and messaging would meet population needs. Student feedback reminded us to clarify further how new content on emerging technologies is grounded in these same health promotion development principles and can be applied in public health settings. Some students questioned how knowledge about these technologies would translate to future work, possibly considering public health settings with limited resources. As part of the “Innovator’s Dilemma” (37), early innovations offer minimal value to customers but compound in value later. Similarly, it may be that courses on innovations, as we instructed, offer greater value to students over a longer time horizon than what some students focus on, like imminent job searches.

Students also wanted to understand the application of these technologies for diverse populations, such as individuals who are deaf, blind or have other accessibility issues. For example, reception is an essential gateway for patient care from small clinics to large hospital systems. LLM chatbots have the potential to alleviate staffing shortages to coordinate patient scheduling and queries (38). AR smart glasses are a viable alternative to video remote interpreting to optimize communication for deaf patients in healthcare settings (39). By adjusting the course to include more clinical and public health case studies like these two examples, students may be able to form more connections between health communication principles, the course content, and their future careers as health professionals developing technology-driven interventions across diverse health settings and populations.

### 4.1 Limitations

Given the recent roll-out of the new course content, our conclusions are based on a single cohort of students. A larger sample size is needed, especially with more representation from students in clinical fields of study, to inform additional curriculum changes and if the additional content on digital health interventions should be expanded to comprise its own course. The results also need to be interpreted considering potential self-selection bias; 69 % of the students completed the course evaluation and only half of the students completed the supplemental evaluation. At least there appeared to be a comparable gender composition between all enrolled students and the subset of students who completed the supplemental evaluation. The recent roll-out of the new content also meant we could only evaluate its perceived value as a short-term outcome Table 2. We could not evaluate actual value as a long-term outcome that would best be evaluated by feedback from alumni who took the class and are developing health campaigns and interventions in the field.

### 4.2 Future research

The current study assessed students’ technology utilization before course enrollment to add context to the appropriateness of the course content for its intended audience. Future research should also assess students’ technology readiness (30) to help educators better evaluate how to optimize course content development and delivery based on students’ comfort and skill levels with different technologies. The current course introduced students to emerging AI-driven technologies that public health researchers are using to develop digital health interventions. Given the proliferation of these technologies, educators also need to develop course content to guide students in the selection and evaluation of digital health intervention technologies. Traditional evaluation methods for randomized controlled trials need to be supplemented with implementation science approaches to account for digital health intervention deployments beyond the confines of controlled experiments (40, 41). Proliferating AI-driven technologies also call for educators to make strategic decisions as to which technology companies they partner with that will supply technologies and software packages for educators to teach students to use in the classroom (32). Costs will continue to be a consideration but will need to be considered in concert with other factors like sustainability in an academic setting as technologies evolve.

## 5 Conclusion

The authors added new course content on AI-driven health intervention technology, including chatbots and AR/VR, to a graduate-level health communication course in a school of public health. The study evaluated students perceived benefits about the new course content using questionnaire survey data. Study findings were mixed as students’ excitement to learn about emerging technologies was tempered with concern. For example, some students wanted more information on chatbots, while others questioned how knowledge about chatbots and AR/VR would strengthen their future roles in public health fields. The main takeaway from the student feedback was to include more public health case studies across diverse populations in the course that incorporate chatbots, AR/VR, and other AI-driven technologies. Reinforcing the relevance of these technologies for public health professionals can increase students’ interest in the new course content. By helping students think beyond the current state of technology in healthcare and public health, instructors can better empower students to become tomorrow’s innovators and leaders in the health professions.

## Data Availability

The raw data supporting the conclusions of this article will be made available by the authors, without undue reservation.
