# Supplementary material for: Empowering tomorrow’s public health researchers and clinicians to develop digital health interventions using chatbots, virtual reality, and other AI technologies
Source: Front Public Health. 2025 Jul 8;13:1577076. doi: 10.3389/fpubh.2025.1577076 (PMC12279801; doi:10.3389/fpubh.2025.1577076)
Supplement: Supplementary file 1 [file Data_Sheet_1.docx]

| **Supplementary Table 1** Email responses from seven faculties queried on whether they knew of courses on digital health intervention development and saw a need for that type of course | |
| --- | --- |
| **Institution** | **Email response** |
| Kent State University | “I’m considering designing a course like this for our PhD program.” |
| New York University | “I think this is a great idea.” |
| University of California, Irvine | “I don’t know of specific classes on this topic, but in the Winter this year I’ll be teaching a special topics grad[uate] class on this topic.” |
| University of California, Los Angeles | “That is super interesting and I think that we do not do a very good job with this.” |
| University of California, Los Angeles | (Last author) There were multiple email exchanges that culminated in the new course content presented in this manuscript. |
| Wake Forest University | “I think there is much need for training in the area of digital health intervention development. We don’t have coursework.” |
| Wake Forest University | “I was a learn this on-the-fly person, but developing courses in this area sounds amazing.” |

| **Supplemental Table 2** Responses to first and second free-response questions in the supplemental evaluation |
| --- |
| **What did you like most about the lectures?** |
| I think this class allows for good discussion because everybody had different experiences with different media platforms and different public health interest. So the lecture material led to great discussions that helped me better understand different concepts. |
| I appreciated the expertise of the professors and their clear knowledge of the subject. |
| There was a lot of topics covered and great resources included in the lectures that I found very useful referencing later in the class as I was working on the projects. |
| I appreciated the diversity of information provided---I felt like each subject was covered in a clear and detailed way. |
| Current referenced research |
| I appreciated learning about different health communication methods |
| Relevant content. |
| Straightforward |
|  |
| **What suggestions do you have for how the lectures could be improved?** |
| Maybe more connection to public health. I think it was helpful to see other connections in the world but seeing examples of more updated and concerning public health topics would be good. Emphasis on having more relevant/updated data. |
| I think some simple logistical items like time of day that the class is held and better lighting in the room would help a lot, as a three hour afternoon slot in a dim room made it difficult to focus on lecture content, instead I often just felt sleepy. Lectures and labs would also benefit from better delineation between the two, such as having separate sections for lab and lecture each week rather than just one three hour block. |
| There were a lot of slides! I loved all the resources though, so it’s a trade off. Maybe there’s a way to cut down but still keep resource slides around? And integrate more small group discussions about the content in class. |
| I think incorporation of more interactive activities, like the website resource search we did, could make the lectures more engaging. |
| Have students present more |
| Having more discussions rather than just having lectures |
| It was hands-on and we were given the information on operating the software. |
| Less lecture more practical application, use of case studies |

| **Supplemental Table 3** Responses to third and fourth free-response questions in the supplemental evaluation |
| --- |
| **What did you like most about the labs?** |
| I really enjoyed the labs. It was a great opportunity to have hands on learning and assistance right then and there. I liked all the different forms of media we explored. The instruction handouts were also helpful especially when having to go back to reference the steps. I also liked how there were examples from past classes for us to refer to. |
| I liked that we learned real skills, and the printed handouts and additional resources were really great to refer to, and are something I will hold onto for future use. |
| Loved getting exposure to all the different applications that are out there, especially the open source ones. It made me more comfortable exploring them knowing that they were recommended by the class. |
| I really liked that we were given the opportunity to practice using the software tools and the thought put into choosing software that is free/easily accessible. |
| Hands-on |
| Being exposed to different media platforms |
| It was hands-on and we were given the information on operating the software. |
| practical and real world application |
|  |
| **What suggestions do you have for how the labs could be improved?** |
| Using more updated and user friendly platforms. ShotCut, audacity, Scribus, WordPress all felt outdated and not very easy to navigate. I think learning Adobe Photoshop, Canva, and other website platforms would have been more helpful and applicable (because i have heard of PH orgs using these platforms in the real world). |
| I mentioned this before, but I think splitting lab and lecture sections up could help a lot. Shorter but more frequent classes helps with attention span, and can also help prevent lecture from bleeding into lab time. |
| For me the hardest part about the labs was the size/layout of the classrooms. I would have preferred a more spread out classroom that would allow the instructors to walk through the class to see how we’re doing. Also, it would be difficult to progress on my own computer and watching what Dr. Lang would do on screen. Leaving more time for labs and letting us try it out on our own/with each other using the directions would, in my opinion, be more effective. Often times I got frustrated not being able to find the right button and stop trying on my own. Having us work together and do it on our own would be a more effective learning experience, and then having the instructors around to ask questions. |
| The lab sessions sometimes felt a little rushed, so I think providing the instructions before class to allow students to familiarize themselves with the activity/software could be helpful. |
| Sometimes, applications did not work the same for all users |
| Having mini activities where we could play with different media platforms (ex: when using Audacity, we could have a mini activity where we'd create a 1 minute long podcast, so we can have more creative, hands-on experience using these platforms) |
| Give students a chance to try the software out on their own as a pre class assignment. |
| more in depth practice |

| **Supplemental Table 4** Responses to fifth free-response question in the supplemental evaluation |
| --- |
| **Were there topics in CHS 292 that you would have liked the instructors to cover in greater or less detail? If so, which ones and why?** |
| There were some topics that I felt we spent too much time on and some not enough time on. I think incorporating more P theory in each lesson would be helpful for us to make important connections to target pops, behaviors, and health communication. Other than that, the material felt pretty balanced. |
| Lecture content could be improved by focusing more on the general principles of health communication, and also by spending much less time on the "newer" technologies that aren't as frequently used or easy to learn. The class would have been more valuable if we spent less time discussing things like virtual reality, games, wearables, etc. They can definitely still be mentioned, but only for a portion of a lecture rather than multiple weeks worth of content. Learning skills and principles with more conventional media makes more sense for a foundational class like this, and are more useful for people looking to work on health communication materials for work. I understand that it is interesting to talk about new technologies, but learning how to make a quality brochure or website is a more marketable and applicable skill than learning about VR. |
| Everything was really in depth, so there’s not much I would change about content specifically. Maybe if there was some assignment or activity where we bring in a health communication that we had seen in our daily life or on campus and we can have discussions about them, pros and cons of the design/message/etc., would be cool. That way there’s more student engagement and it feels relevant to our lives and UCLA community. |
| [Left blank] |
| Greater detail on social media |
| More discussion about social media platforms such as TikTok, Instagram |
| More about chatbots - this is an up an coming area. Bringing in guest speakers would also have been engaging. |
| More adobe use |
| **Were there topics in CHS 292 that you would have liked the instructors to cover in greater or less detail? If so, which ones and why?** |

| **Supplemental Table 5** Responses to sixth and seventh free-response questions in the supplemental evaluation |
| --- |
| **Were there labs in CHS 292 where you would have liked the instructors to have spent more or less time providing instruction on how to use the software? If so, which ones and why?** |
| More time on websites, podcasts, animation video, and social media post development. Less time on ShotChut and Scribus. The chatbot was nice and important bu easy and maybe we could have learned another platform during that lab. |
| More time on scribus or similar publishing software would be helpful, because those basic skills with print media can be applicable to so many different other health materials. Chatbot development was interesting, but maybe not the most valuable. Since there is such limited class time I would maybe offer that as a supplemental resource and not have it take up class time. |
| I think for the most part all the labs could have had less active instruction on how to use the software. I would Havel liked it better if we had just been given the instructions and the end goal of the lab and be able to attempt it for the last hour or so individually/in groups. |
| [Left blank] |
| More time on how to use it. |
| Having labs where we used more popular media platforms (ex: CapCut, Photovoice, Camtasia, Storyline, Captivate) |
| Just more time for us to learn how to use it. |
| More instructions on photo editing |
|  |
| **Are there other health communication-related topics that were not covered in CHS 292 that you would have liked to have been covered? If so, which ones and why?** |
| Forms of media that populations that have accessibility issues (deaf, blind, etc.) and how we can reach these populations through health communication. |
| Not necessarily, I think just more time on design basics could have been good. I love design, so I don't need that foundational info, but I think it would have been helpful for the class to spend more time on the general principles of color, font, accessibility, readability, etc etc. |
| I would love to see more examples across different communities. We did see health communication examples in class, and we discussed the different ways that might be more effective for different communities, but seeing those examples would have been interesting. |
| [Left blank] |
| No |
| How to combat health misinformation/disinformation |
| N/A |
| None |
